# The overlooked evolutionary dynamics of 16S rRNA revises its role as the “gold standard” for bacterial species identification

**DOI:** 10.1038/s41598-024-59667-3

**Published:** 2024-04-20

**Authors:** Oldřich Bartoš, Martin Chmel, Iva Swierczková

**Affiliations:** 1 Military Health Institute, Military Medical Agency, 16200 Prague, Czech Republic; 2 Department of Infectious Diseases, First Faculty of Medicine, Charles University and Military University Hospital Prague, 12108 Prague, Czech Republic

**Keywords:** Evolutionary biology, Microbiology

## Abstract

The role of 16S rRNA has been and largely remains crucial for the identification of microbial organisms. Although 16S rRNA could certainly be described as one of the most studied sequences ever, the current view of it remains somewhat ambiguous. While some consider 16S rRNA to be a variable marker with resolution power down to the strain level, others consider them to be living fossils that carry information about the origin of domains of cellular life. We show that 16S rRNA is clearly an evolutionarily very rigid sequence, making it a largely unique and irreplaceable marker, but its applicability beyond the genus level is highly limited. Interestingly, it seems that the evolutionary rigidity is not driven by functional constraints of the sequence (RNA–protein interactions), but rather results from the characteristics of the host organism. Our results suggest that, at least in some lineages, Horizontal Gene Transfer (HGT) within genera plays an important role for the evolutionary non-dynamics (stasis) of 16S rRNA. Such genera exhibit an apparent lack of diversification at the 16S rRNA level in comparison to the rest of a genome. However, why it is limited specifically and solely to 16S rRNA remains enigmatic.

## Introduction

Ever since 1977, which was a breakthrough year for the invention of the so-called first-generation or Sanger sequencing^1,2^, the attention of leading evolutionary biologists and taxonomists has been focused on 16S rRNA^3,4^. 16S, 23S and 5S rRNAs are essential genes that typically constitute a chromosomal rRNA operon^5^. It is the only component that is universal to all self-replicating organisms and its sequences change only slowly over time^3^. Prior to these advances, it was practically impossible to achieve any valid phylogeny, especially in microbiology, but the advent of both sequencing techniques and the discovery of the potential of 16S rRNA essentially changed the world of microbiology^6^. Since then, (not only) microbiology has built on and, in many ways, relied on 16S rRNA as a universal and reliable marker for species identification and delimitation^7–13^.

16S rRNA is also widely used in clinical practice, where it has served as a powerful tool for bacterial identification and diagnostics for decades^14,15^. Although today's clinical practice uses preferentially Mass Spectrometry for diagnostic purposes, especially MALDI TOF instruments^16,17^, sequencing procedures targeting 16S rRNA remain an important part of the portfolio of microbiology laboratories^15^. This is because while MALDI TOF usually provides fast and correct identification, in a significant number of cases it fails to provide any valid information^15,18^. Whereas 16S rRNA sequencing always gives us at least some idea of the phylogenetic classification of a given organism/pathogen^15^.

The reason we began to doubt the specificity of 16S rRNA was the routine identification of an unknown fish pathogen. Both types of analyses were performed, but while the 16S rRNA classification quite clearly identified the sample as a common bacterial species, the MALDI TOF classification failed. We decided to resolve this apparent discrepancy by whole-genome sequencing and detailed characterization of the sample. It turned out as a new species whose average nucleotide identity (ANI) of the genome with the nearest described species was only about 82.5%, while the threshold for describing a new species is reported to be around 95%^19^. Therefore, we started to investigate why two evolutionarily well-separated entities essentially share the same copy of 16S rRNA.

Despite that 16S rRNAs are thought to be species-specific^20^, and the assumptions that genes/molecules involved in complex interactions (such as ribosomes) should not be subject to Horizontal Gene Transfer (HGT)^21^, several studies have reported cases of HGT of 16S rRNAs and some have even evaluated the (in)significance of this phenomenon on the viability of a given organism^20,22,23^. Particularly for this type of sequence, which is typically found in multiple copies in a genome, the availability of a complete genome sequence is crucial for HGT evaluation, which has been facilitated by the advent of third-generation sequencing technologies and platforms^24^. On the other hand, difficult-to-detect HGT within a bacterial species or between closely related species is probably an important driving force in the evolution of microorganisms^25^. Recently, some studies have emphasized that 16S rRNA, especially in a phylogenetic reconstruction/estimation context, provides inaccurate results, suggesting the involvement of HGT^26–28^.

Here we show that the relationship between 16S rRNA and species delimitation and classification is likely to be more complex than previously reported. Furthermore, we focus on the question of why and how it actually resists something that, especially in biology, is considered to be one of the basic definitions of life, i.e. change/evolution^29^. From this point of view, it seems more appropriate to consider them rather as living fossils carrying information about the origin of the domains of cellular life^4,30–32^. Further, the role of functional constraints on the evolutionary rigidity of 16S rRNAs has been largely refuted by the study of mitochondrial 16S rRNAs, whose evolutionary dynamics does not significantly differ from those of typical nuclear genes^33–35^. Therefore, we studied and evaluated the evolutionary dynamics of 16S rRNA in more than 15 bacterial genera comprising over 1,200 species. Further, we also extended this analysis to some representatives of the Archaea and Eukaryotic domains. Next, we documented the manifestations of intra-specific evolutionary dynamics of 16S rRNA in a well-studied species, *Escherichia coli*, whose explanation requires the effective action of HGT and/or concerted evolution.

## Results and discussion

### The performance of 16S rRNA as a marker for the identification of bacterial species

**Figure 1:**
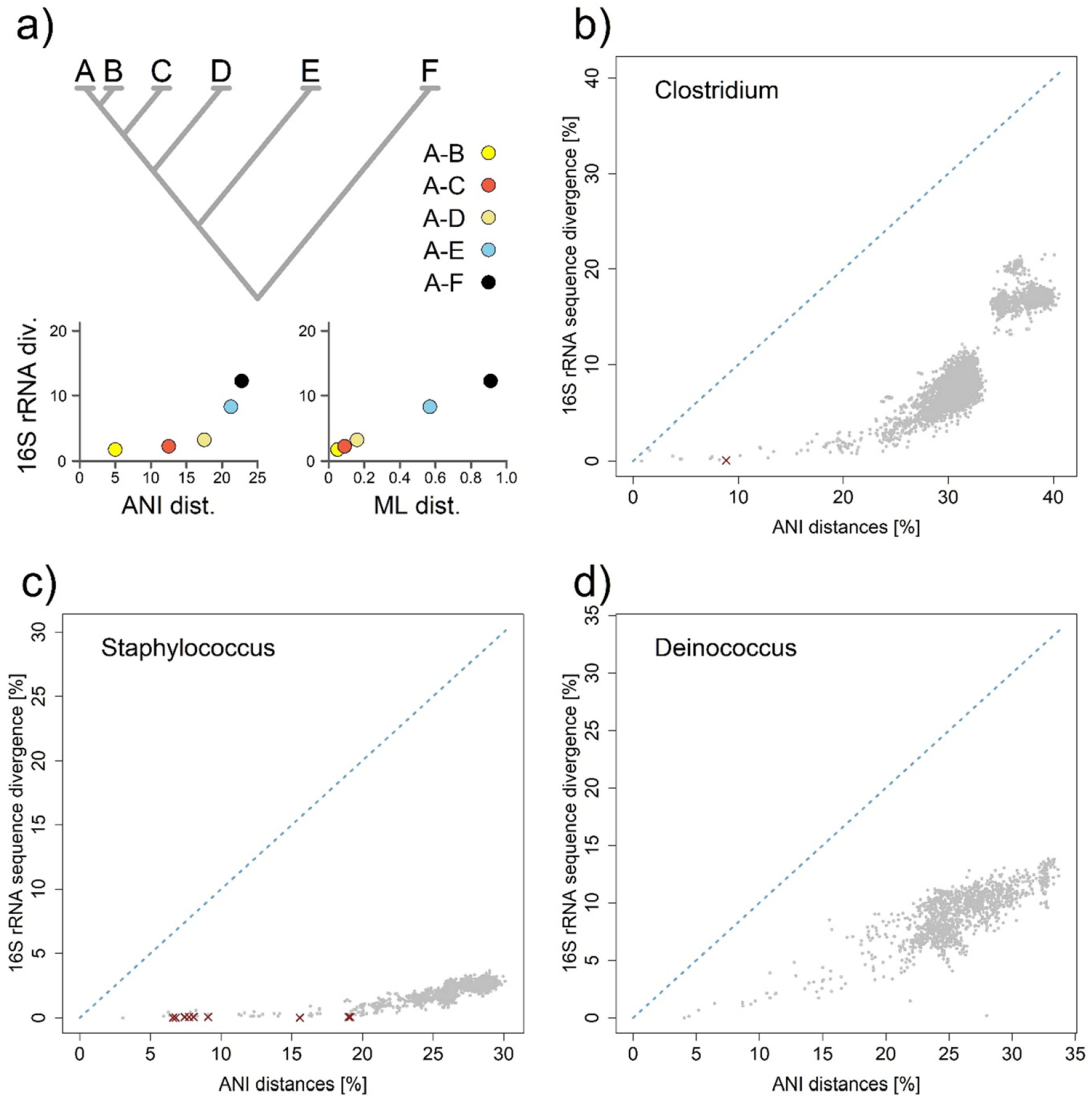
Comparison of evolutionary (substitution) rates in selected bacterial genera as a function of Average Nucleotide Identity (ANI) estimates: (**a**) schematic representation of a phylogenetic tree with varying degrees of evolutionary distances to the hypothetical species A. All species pairs involving species A are shown in illustrative plots comparing 16S rRNA sequence divergence in response to genomic divergence. We measured genomic diversity in two different ways, using either average nucleotide identity (ANI) or maximum likelihood (ML) phylogenetic distances. While ANI is useful for distinguishing (relatively) closely related species or isolates, whereas ML distances are a more appropriate measure for macro-evolutionary scales. (**b**) Clostridium; (**c**) Staphylococcus; (**d**) Deinococcus. Individual species-to-species comparisons are shown in gray, dark crosses indicate species pairs that share essentially the same copy of 16S rRNA (identity > 99.9%), despite being evolutionarily well-separated entities. Graphs of all taxa studied can be reviewed in Supplementary Fig. 3.

First, to gain insight into how 16S rRNA corresponds to overall divergence at the genome level, we estimated a matrix of evolutionary distances within each single bacterial genus. We used two different methods to estimate evolutionary distances for pairwise comparisons between individual species (see Fig. 1a). First, we used average nucleotide identity (ANI), which is a useful estimate especially for discrimination of closely related species/lineages^19,36^. In contrast, protein-based phylogenomic Maximum Likelihood distances are more useful for disentangling and inferring deeper evolutionary relationships^37^. We initially focused on how and whether divergence at the 16S rRNA level actually reflects species boundaries, defined as ~ 95% divergence in the genome sequence.

To our surprise, immediately after the analysis of the first bacterial genus, it was apparent that the divergence of 16S rRNA between "relatively" closely related species is an essentially non-existent phenomenon (see Fig. 1b, c). Moreover, we instantly identified a case where two species shared a basically identical 16S rRNA variant (> 99.9% identity), although at the whole genome level they clearly represented evolutionarily well-separated entities (see Fig. 1b). We show that this is in fact not as rare a phenomenon as one would assume, however, beyond this we show that individual genera show significant differences in the evolutionary dynamics of 16S rRNA (see Fig. 1d). We analyzed 15 major bacterial genera comprising over 1200 species (see Table 1), with each species represented by only one reference sequence (see Supplementary Table 1). Despite such a limited scope, we were able to detect over 175 such cases where two well-differentiated species possess essentially identical copies of 16S rRNA. These analyses were performed within individual bacterial genera, but when we similarly analyzed the data on an inter-generic scale, we found no clear evidence to suggest any recent HGT. These results generally question, at least to some extent, the applicability of 16S rRNA as a species-specific or even strain-specific marker^9^, as well as its suitability for phylogenetic reconstructions of closely related taxa^12^.

**Table 1:** List of genera/taxa included in this study.

Genus/taxon	Species	HGT pairs	16S rRNA copies	SCO genes
*Clostridium*	102	1	4 (1; 9,5)	86
*Streptococcus*	106	1	4 (1; 5)	311
*Bacillus*	107	30	4,5 (1; 10)	98
*Corynebacterium*	137	0	4 (1; 4)	197
*Bartonella*	43	2	2 (1; 2)	369
*Staphylococcus*	57	9	6 (5; 6)	795
*Burkholderia*	44	7	4 (1; 6)	1141
*Rhizobium*	80	52	1 (1; 3)	804
*Acinetobacter*	83	0	4 (1; 7)	756
*Mycobacterium*	119	9	1 (1; 1)	421
*Vibrio*	123	1	8 (1; 11)	245
*Nocardia*	99	2	1 (1; 1)	535
*Leptospira*	69	62	1 (1; 2)	1068
*Deinococcus*	54	0	1 (1; 3)	518
*Legionella*	16	0	4 (3; 4)	404
Subtotal	1240	176	NA	NA
*Thermococcus*	31	3	1 (1; 1)	720
*Aves*	41	0	NA	147
*Actinopterygii*	35	0	NA	115
Total	1348	179	NA	NA

For each taxon we report: the number of species; the number of detected horizontal gene transfers (HGT), i.e. species pairs sharing the same variant of 16S rRNA (> 99.9% identity); median of copy number of 16S rRNA with interquartile ranges; number of detected single-copy orthologous (SCO) genes used to estimate the phylogenetic distances.

### The rate of evolution of 16S rRNA

The rate of 16S rRNA evolution between relatively closely related species, i.e. ranging from 5% up to 20% divergence, is generally extremely low compared to other genes or the rest of the genome as a whole. 16S rRNA is truly exceptional due its ability to at least seemingly resist something that is perfectly natural in terms of evolutionary biology, namely change^29^. Virtually identical evolutionary trends can also be observed for 23S rRNA (see Supplementary Fig. 1), but for simplification and its uttermost significance, we will consider only 16S rRNA for the purposes of this text. But how is it possible that this particular sequence maintains a significantly lower mutation rate than the rest of the genome? For a long time, evolutionary biology has been concerned with the concept of Essentiality, which suggests that essential genes, such as 16S rRNA, should evolve slower than more dispensable ones due to increased selection pressure^38^. However, until recently, attempts to confirm this theory have remained ambiguous, suggesting quite clearly that so-called Essentiality affects the rate of evolution in no fundamental way^38,39^. Even in this perspective, it is clear that 16S rRNA represents an entirely exceptional example in its evolutionary rigidity.

But what does this rigidity stem from? When, on the one hand, it has been experimentally proven that bacteria can tolerate both recombinant and even foreign copies of 16S rRNA without much difficulty^20,23^. On the other hand, the 16S rRNA, which is encoded by the mitochondrial genome and is the basis for the formation of the mitoribosome and thus retains the same function as its bacterial counterpart, lacks this unique characteristic^33,34^. Actually, mitochondrial 16S rRNA even lacks some otherwise conserved structural elements whose role has been taken over by ribosomal proteins encoded in the eukaryotic genome^35^. Perhaps most interesting is the fact that nuclear mito-ribosomal proteins mirror the increased evolutionary rate of the mitochondrial genome. As a result, nuclear mito-ribosomal proteins evolve more than 10 times faster than cyto-ribosomal proteins, despite being part of the same nuclear genome^33–35^. These facts show quite clearly that the Essentiality along with the assumed evolutionary constraints it implies do not in themselves provide a satisfactory explanation of the observed phenomenon, i.e. extremely slow evolution rate of 16S rRNA.

### Differences between bacterial genera

**Figure 2:**
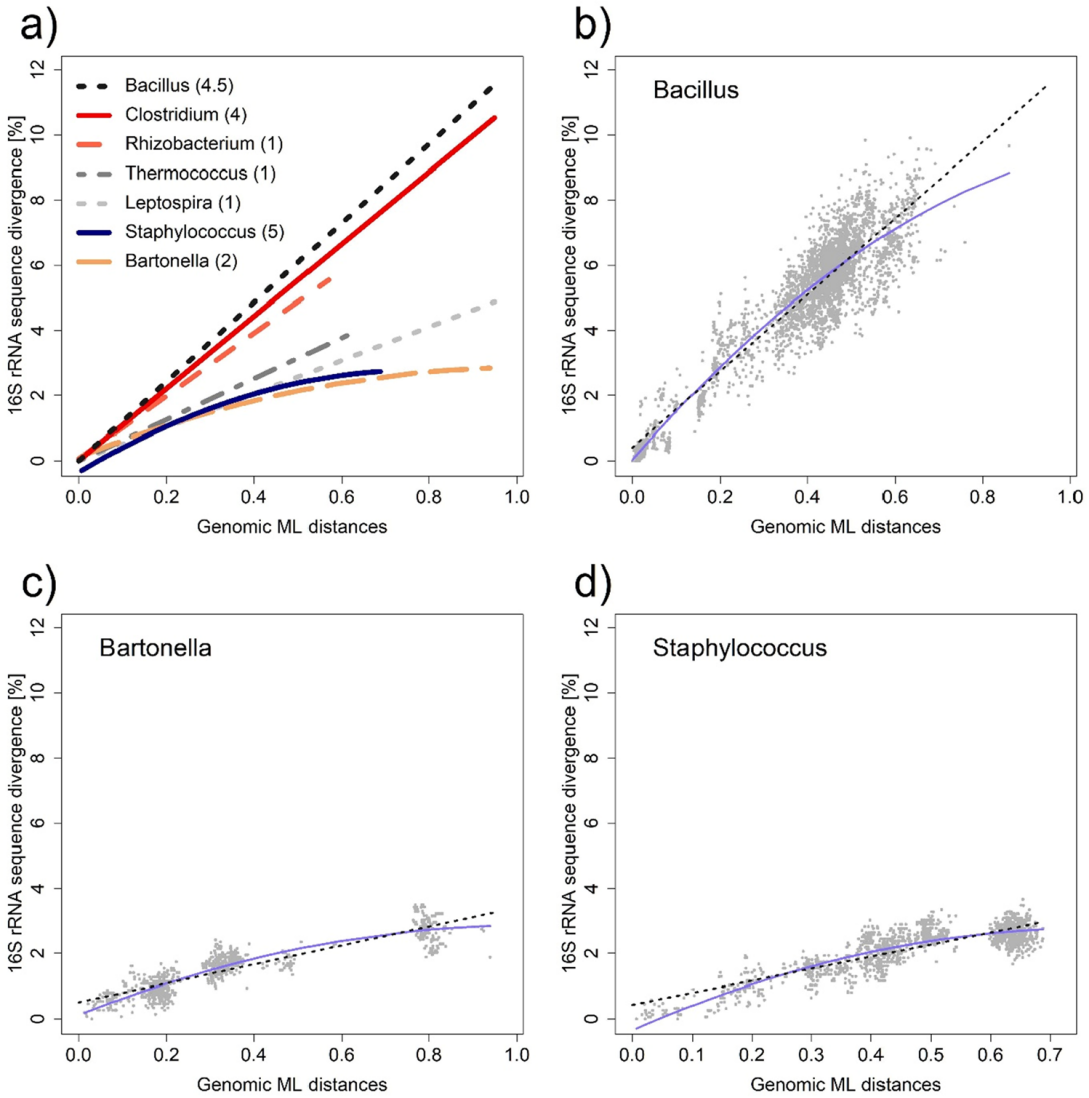
Comparison of evolutionary (substitution) rates in selected bacterial genera as a function of genomic maximum likelihood (ML) evolutionary distances: (**a**) comparison of selected (bacterial) genera, each represented by a linear regression or second order polynomial model. For all linear regressions in this graph, we deliberately set the intercept to zero for better readability. We preferred the polynomial regression model where it fit the data significantly better than linear regression, both statistically and especially visually. Numbers in parentheses represent the median 16S rRNA copy number for a given bacterial genus. Note that we did not observe any apparent difference in the rate of mutation accumulation at the level of 16S rRNA between genera with higher or lower copy number of this sequence/gene. (**b**) Bacillus, (**c**) Bartonella, (**d**) Staphylococcus. Individual species-to-species comparisons are shown in gray. The black dashed line represents the fitted linear regression model, while the blue solid line represents the second-order polynomial function/model. Graphs of all taxa studied can be reviewed in Supplementary Fig. 4.

One of the unique features of 16S rRNA is that it is typically found in multiple copies per genome. Copy number is usually a relatively stable characteristic of a given genus, but there are notable differences between genera, and a typical bacterial species such as *E. coli* contains about 7 copies on average^40^. In theory, genes with multiple copies, such as 16S rRNA, should be subject to significantly stronger negative selection than single-copy genes^41^. Thus, we tested whether the rate of 16S rRNA evolution depends on the number of copies of 16S rRNA contained in the average species of each genus. In this comparison, we also included the prokaryotic genus Thermococcus, which is the only one of the selected genera to contain only a single copy of 16S rRNA. Unlike the first analysis, where we were mainly interested in separating and visualizing closely related species around the imaginary (5%) species boundary, this time we used Maximum Likelihood (ML) distances, which are a more reliable measure especially in relation to deeper phylogenetic relationships (see Fig. 2). Interestingly, it turned out that the copy number of 16S rRNA has no obvious effect on the rate of its evolution (at least in bacteria) (see Fig. 2a), but it led to two somewhat unexpected findings.

The first and more obvious finding is that there are significant differences between the genera, but they do not correspond at all to the copy-number hypothesis, which is in line with an earlier study^42^. The second finding is that, despite quite reasonable expectations, we have observed a rather significant deviation from the linearity of mutation accumulation (evolution) over time in some genera (compare Fig. 2b with c, d). The expectation of linearity follows both from the so-called Neutral theory of molecular evolution^43,44^ and from actual experimental data^39,45^. Since these are very conserved sequences, the expected linear relationship cannot be disrupted even by substitution saturation^46^. We suggest that the most likely explanation for this phenomenon, barring a mistake, may be a relatively high level of elusive HGT^42^, especially among closely related species, which stabilizes a particular 16S rRNA phenotype/genotype at the genus level. This is particularly evident in the aforementioned prokaryotic genus Thermococcus, which, despite expectations, shows one of the lowest diversifications at the 16S rRNA level. And it is mainly extremophilic organisms in which HGT is often mentioned not only as a means of adaptation, but especially as a necessity for maintaining genome integrity^25,47^. In general, it is known that the susceptibility of different bacterial genera to HGT varies and is probably related to their life-history traits^48^.

### Comparison of bacteria with vertebrates

To assess the potential role of HGT, we needed to obtain data from organisms for which it can be ruled out a priori. We therefore chose representatives of vertebrates, i.e. fish (Actinopterygii) and birds (Aves), which are characterized by relatively small genomes^49^. Unlike bacteria, vertebrates have two functional equivalents of bacterial 16S rRNA, the first being eukaryotic cytoplasmic 18S rRNA and the second being mitochondrial 16S rRNA.

**Figure 3:**
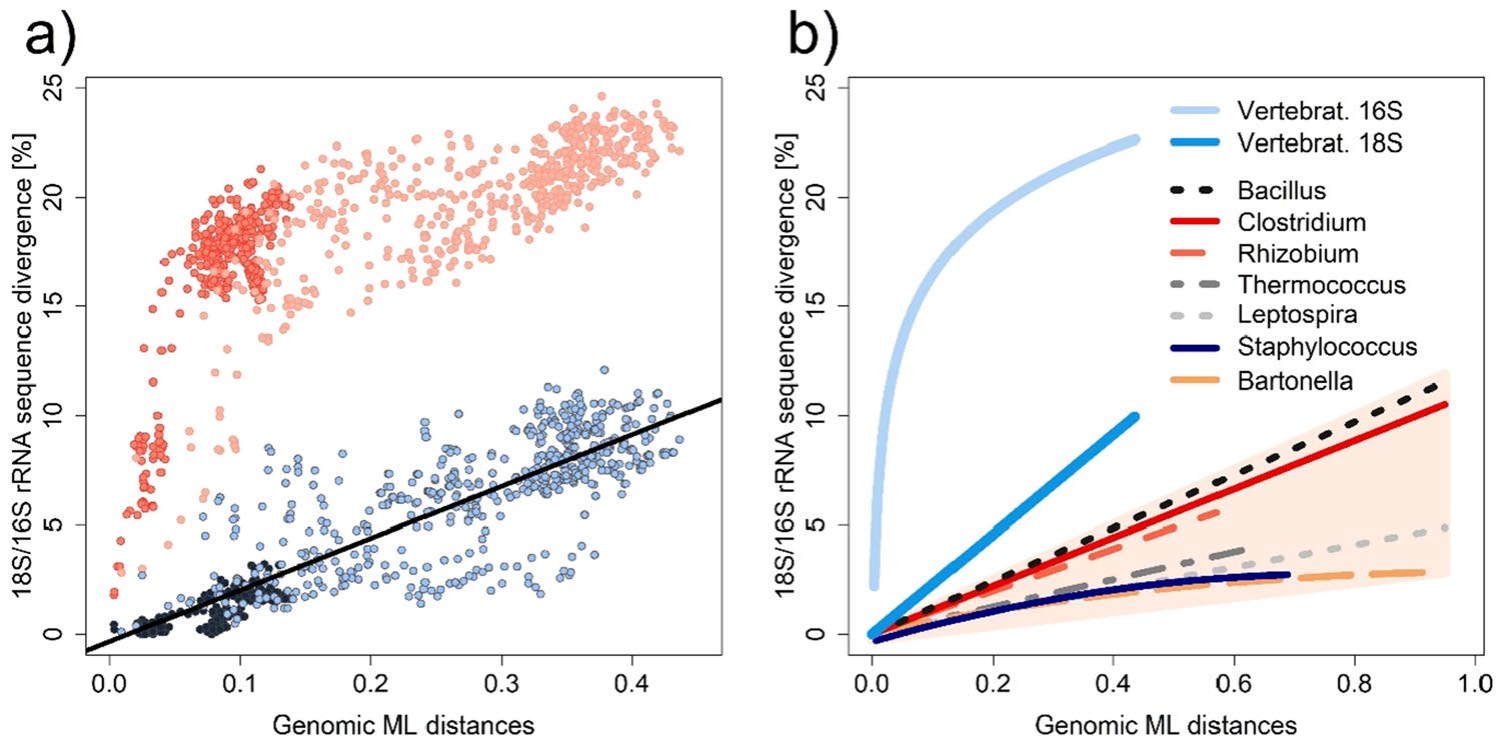
Comparison of evolutionary (substituton) rates between vertebrate eukaryotic 18S rRNA and mito-ribosomal 16S rRNA: (**a**) Data for 18S rRNA are shown in shades of blue, whereas mito-ribosomal 16S rRNA are shown in shades of red. The darker shade always represents the group of birds (Aves), while the lighter shade represents the group of selected fish (Actinopterygii). Linear regression is shown for the 18S rRNA data (black). The data are shown separately in Supplementary Fig. 5; (**b**) comparison of evolutionary (substitution) rates between selected bacterial genera and vertebrate eukaryotic organisms, respectively their 18S rRNA and mito-ribosomal 16S rRNA. The bacterial genera are the same as in Fig. 2a and are shown on a beige background. Eukaryotic 18S rRNAs are represented by the linear model in bright blue, whereas mito-ribosomal 16S rRNAs are represented by a logarithmic regression model shown in light blue.

In contrast to some bacterial genera, the evolutionary (substitution) rate of 18S rRNA gene of the vertebrates follows the expected linear model (see Fig. 3a), i.e. linear regression fits the data better than a second-order polynomial model. The evolutionary rate of mito-ribosomal 16S rRNA is much higher and the only thing that slows it down, at least optically, is substitution saturation (see Fig. 3a). Figure 3b shows an overall comparison of 16S/18S rRNA evolution models from selected bacterial genera as well as data from vertebrates. In summary, in organisms for which we have ruled out a priori the effective action of HGT, we can state that evolution (mutation accumulation) proceeds linearly in evolutionary time, i.e. in accordance with the theoretical expectations. Quite surprisingly, the substitution rate of 18S rRNA is higher than that of the bacterial genera examined, despite the fact that 18S rRNA is considered to provide only low-level taxonomic resolution in vertebrates^50^. Instead, mitochondrial rRNA is often used for accurate identification at the species level^51^.

### Intraspecific 16S rRNA evolutionary dynamics

The most convincing evidence of HGT and/or concerted evolution was obtained by analyzing ~ 3700 'complete' genomes of *Escherichia coli* isolates. We found that there are at least three distinct 16S rRNA variants/genotypes within these genomes that possess the following key characteristics: (1) they are clearly distinguishable from each other; (2) they are not linked to specific phylogenetic lineages; (3) and, most importantly, we are able to find genomes in which only one variant is represented, but also those in which these variants are combined in different ways.

**Figure 4:**
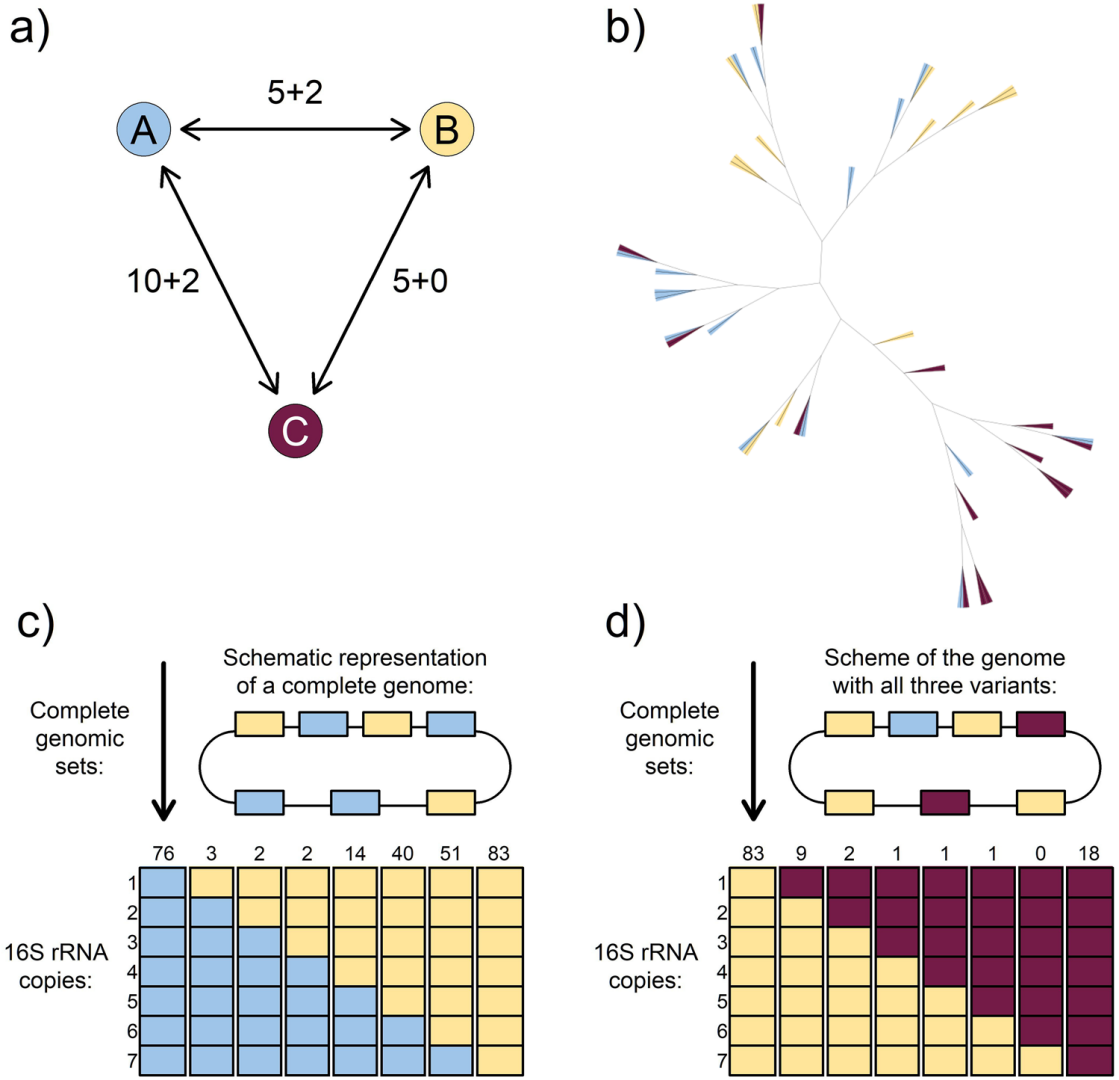
(**a**) Schematic representation of discovered variants of 16S rRNA in *Escherichia coli* genomes. The numbers along the arrows indicate the number of mismatches + gap openings. (**b**) Schematic phylogenomic tree of *Escherichia coli* genomes in which exclusively one of the 16S rRNA reference variants was identified. (**c**) Schematic representation of detected genomes with mixed representation of variants A and B. Numbers above the columns indicate the number of detected genomes with a given ratio/number of variants. (**d**) Schematic representation of detected genomes with mixed representation of variants B and C.

We screened thousands of genomes and looked for variability within 16S rRNA copies of individual genomes. In most cases, the variability assessed by BLAST resulted mainly from deletion-affected and hence possibly non-functional copies. However, within *E. coli* isolates, we thus revealed that these genomes host at least three well-defined 16S rRNA variants (see Fig. 4a). We selected three groups of representative genomes for which we were able to confirm that all (seven) copies of 16S rRNA clearly matched only one of the identified reference variants. We then estimated a phylogenomic tree that clearly showed that the possession of one or another variant is essentially randomly distributed across the entire phylogeny (see Fig. 4b). Therefore, we can exclude that these are specific variants linked to phylogenetically lower taxa, e.g. subspecies. While the simple presence of multiple variants could be explained, for example, as Incomplete Lineage Sorting (ILS)^52^, the presence of genomes/strains with(out) a mixture of variants cannot be satisfactorily explained without the involvement of HGT or efficient concerted evolution. In fact, we identified virtually all possible combinations of the full genome set of 16S rRNA copies involving a combination of variants A and B, as well as B and C (see Fig. 4c, d). Although in this case we cannot rule out that this is intraspecific variation and not a manifestation of HGT, these data demonstrate the power and importance of concerted evolution for the evolutionary dynamics of 16S rRNA.

In fact, this variability at the 16S rRNA level has been described previously and its significance appears to be biologically relevant^53^; but see^54^. In particular, transcriptional upregulation of a variant referred to as rssh 16S rRNA (referred to here as variant B) is associated with a general stress response, activation of starvation related gene pathways and increased antibiotic resistance^53^. While transcription and not translation is considered the major controlling element of gene expression, it turns out that even translation and therefore ribosomes as such can play an important regulatory role^53,55^.

The rssh 16S rRNA variant has been described in the model strain K-12, in which it typically occurs only in single copy^53^. Therefore, we examined genomic metadata deposited at NCBI to determine whether the documented accumulation of copies of this particular 16S rRNA variant is associated with any specific location or phenotype. We found that enrichment of this particular 16S rRNA variant is tightly associated with the enterohemorrhagic *E. coli* (EHEC) serotype O157:H7 (see Supplementary Fig. 2), which has been reported in many countries worldwide^56^. EHEC serotype O157:H7 was first recognized in 1982 and is considered a major public health concern as the causative agent of hemorrhagic colitis and life-threatening hemolytic uremic syndrome in humans^56^. Transmission usually occurs through consumption of contaminated food or water, and therefore serotype O157:H7 is considered a food-borne pathogen^56^. Interestingly, serotype O157:H7 has been described as having a survival capacity far exceeding that of common commensal strains, allowing it to survive the harsh conditions frequently encountered in the human food chain^57^.

## Conclusions

In this study, we have clearly demonstrated why it is not reasonable to rely on 16S rRNA as a species-specific marker. Undoubtedly, 16S rRNA remains a valuable marker due to its unique properties, but we must be aware of its limitations. Tools that overcome these limitations already exist today, such as Metagenomic Shotgun Sequencing^58^, but their wider adoption outside the high-end research environment cannot be expected in the near future.

However, we have shown that although 16S rRNA can be considered one of the most sequenced and studied sequences, its unique evolutionary dynamics has long been overlooked. We have demonstrated that both Horizontal Gene Transfer (HGT) and concerted evolution can play a significant role in the evolution of 16S rRNA.

While the effects of HGT on the evolutionary dynamics of 16S rRNA at the inter-specific level seem to be clear, the role of concerted evolution is somewhat ambiguous. In theory, the effective action of concerted evolution at the species level should counteract inter-specific HGT^59^, at least unless other processes such as selection or molecular drive are involved^59,60^. On the other hand, 16S rRNA sequences of closely related taxa are similar to such an extent that their fixation/loss can be driven by genetic drift (chance) alone.

However, why HGT is so remarkably exhibited at the level of 16S rRNA remains enigmatic. Especially considering the fact that we have ruled out that this could be related to its most obvious feature, i.e. its multiple representation in the genome.

## Supplementary Information

Supplementary File 1.

Supplementary Figures.

Supplementary Table 1.

Supplementary Table 2.

Supplementary Table 3.

Supplementary Table 4.

## Data Availability

This study was based on the analyses of publicly available genomic data from NCBI repositories, their accession numbers are provided in Supplementary Tables 2, 3 and 4.
