# Author Correction: The overlooked evolutionary dynamics of 16S rRNA revises its role as the “gold standard” for bacterial species identification

**DOI:** 10.1038/s41598-024-72328-9

**Published:** 2024-09-12

**Authors:** Oldřich Bartoš, Martin Chmel, Iva Swierczková

**Affiliations:** 1 Military Health Institute, Military Medical Agency, 16200 Prague, Czech Republic; 2 Department of Infectious Diseases, First Faculty of Medicine, Charles University and Military University Hospital Prague, 12108 Prague, Czech Republic

Correction to: *Scientific Reports* 10.1038/s41598-024-59667-3, published online 20 April 2024

In the original version of this Article, the Supplementary Information file, which included the Materials and Methods section, was omitted from the Supplementary Information section.

The Supplementary file now accompanies the original article.

## Supplementary Information

Supplementary File 1.

