# Supplementary material for: The overlooked evolutionary dynamics of 16S rRNA revises its role as the “gold standard” for bacterial species identification
Source: Sci Rep. 2024 Apr 20;14:9067. doi: 10.1038/s41598-024-59667-3 (PMC11032355; doi:10.1038/s41598-024-59667-3)
Supplement: Supplementary file 1 [file 41598_2024_59667_MOESM1_ESM.docx]

**Supplementary Materials and Methods**

**Data Acquisition**

All data were obtained from publicly available NCBI databases. The data were downloaded between March and June 2023. For all included bacterial genera, we obtained the genome of all available species that met the following conditions: 'representative genome' and 'complete genome'. A list of the genomes is available in **Supplementary Table 2**. For each species, we obtained genome sequence, coding sequences (CDS), messenger RNA sequences (mRNA) and protein sequences.

We followed a similar strategy for vertebrate taxa, i.e. birds and fish, for which we obtained genome sequences, CDS and protein sequences. However, in contrast to bacterial genomes, 18S rRNA as well as mito-ribosomal 16S rRNA sequences were searched and retrieved manually from NCBI databases. Also, especially for fish, we decided to select an essentially random sample of 35 fish genomes, which was quite sufficient for our purposes, to reduce the computational and logistical burden.

**Bacterial 16S rRNA Sequences Extraction**

These analyses were performed separately for each selected bacterial genus. Within each species (representative genome), we used R script to automatically search all RNAs within the RNA file downloaded from NCBI for the occurrence of the phrase "16S"**^1^**. We used R libraries stringr and seqinr**^2,3^**. The longest sequence was selected as representative of the species/genome if it was longer than 1350 bp. We then used BLASTn to calculate the "all-to-all" percentual sequence dis/similarity**^4^**.

**Estimates of Evolutionary Distances Between Bacterial Genomes**

To estimate the evolutionary distances between individual bacterial species, we adopted two strategies. First, we used the so-called orthologous Average Nucleotide Identity (ANI), a method/estimate that is effectively used today as a measure of novelty/differentiation for identifying new species in microbiology**^5^**. However, this method/estimate is particularly useful for distinguishing closely related species, but mainly due to substitution saturation fails to resolve deeper evolutionary relationships. Therefore, we also used a phylogenomic approach. Using OrthoFinder**^6^**, we identified all single-copy orthologous genes/sequences, ranging from 86 to 1141 depending on the genus and its evolutionary characteristics. Multiple Sequence Alignments were conducted using the software MAFFT v7.505**^7^**. In order to estimate the species tree, we concatenated individual genes using the AMAS tool**^8^**. Maximum Likelihood gene trees were reconstructed by IQ-TREE v2.2.0**^9^** using the extended model selection with a free rate of heterogeneity in combination with 1000 ultrafast bootstrap replicates**^10,11^**. In this way we obtained Maximum Likelihood distances for each pair of two species.

**16S rRNA vs. Genomic/Evolutionary Distances - Horizontal Genetic Transfer Detection**

For better readability, we have converted BLASTn similarity estimates to dissimilarity percentages, as well as ANI estimates. We then plotted these values against the ANI estimates, which allowed us to assess the evolutionary dynamics of 16S rRNA relative to the rest of the genome. Each point in this graph represents one pair of species of a given genus, where the x-axis plots the evolutionary distance between the two species and the y-axis plots the sequence divergence between these species estimated from their 16S rRNA. Similarly, we then used Maximum Likelihood distances instead of ANI estimates. We considered Horizontal Gene Transfer to occur when ANI divergence between species was greater than 5 percent but they carried essentially the same 16S rRNA, i.e. >99.9 percent identity. Only comparisons that provided meaningful alignments exceeding 1350 bp were considered relevant.

**Testing the Dependence of Evolutionary Rate on 16S rRNA Copy Number**

In light of the study by Mano**^12^**, which suggests that genes with multiple copies, such as 16S rRNA, should be subject to significantly stronger negative selection than single-copy genes. We hypothesized that this apparent property of 16S rRNA, i.e. variable copy number on an inter-generic scale, could largely predict its evolutionary dynamics. Therefore, we estimated the average/typical 16S rRNA copy number using BLASTn and custom R scripts for each genus. For the purpose of this analysis, we also included one Archaeal genus in the test set, Thermococcus, which is the only one of all other genera characterized by the fact that its members possess strictly only a single copy of 16S rRNA.

**Testing the Linearity of 16S rRNA Evolution**

To understand what processes influence 16S rRNA evolutionary dynamics, it is important to determine whether apparent sequence evolution deviates significantly from the linear model/expectation. To test this hypothesis, we limited the range of evolutionary distances, defined as Maximum Likelihood distances, to values between 0 and 0.95. This was important in order to avoid the phenomenon of substitution saturation. Then we fitted a linear (regression) model and a second-degree polynomial model for each group (genus). Such a polynomial model allows for deviations from linearity by allowing the line to bend/curve. We then used the aictab function of the R library AICcmodavg to estimate which of these models fits the data better based on the Akaike information criterion**^13^**.

**Assessment of Eukaryotic 16S/18S rRNA Evolution**

We also included vertebrate Eukaryotic organisms in this study because analysis of their 18S rRNA, the functional equivalent of bacterial 16S rRNA, and mito-ribosomal 16S rRNA can help us understand what shapes the evolutionary dynamics of these unique sequences. First, we were interested in the evolutionary dynamics of 18S rRNA sequences in these organisms, in which we basically exclude *a priori* any significant role of Horizontal Gene Transfer (HGT). Also, it allowed us to compare the rate of evolution between 18S rRNA and mito-ribosomal 16S rRNA. In all cases, sequences of a given species were considered relevant only if they were available, but especially if, when compared for a given species pair, BLASTn produced meaningful alignments of at least 1350 and 1550 bp in length for mito-ribosomal 16S rRNA and 18S rRNA, respectively. Comparisons that included the 18S rRNA sequence of *Falco cherrug* showed anomalous behaviour and were therefore excluded from the analyses. Linear regression and Pearson correlation coefficients were calculated for the 18S rRNA sequences**^14,15^**. As above, we tested whether the sequence evolution of 18S rRNA follows a linear trend or not.

**Assessing Intra-specific 16S rRNA Variability**

If HGT is as significant as it appears to be at the intra-specific level, then it would be reasonable to expect its role and frequency to be much higher at the inter-specific level. On the other hand, if we were to consider that the frequency of HGT would be high, in addition to being coupled with effective concerted evolution**^16^**, its effect would be effectively untraceable to us. However, data from the intra-specific level represent the best opportunity to try to capture the effects of these phenomena.

Therefore, we attempted to assess the intra-specific diversity of 16S rRNA in several well-known and particularly frequently sequenced species, i.e. *Staphylococcus aureus*, *Salmonella enterica* and *Escherichia coli*. For which we obtained 1525, 2194 and 3701 genomes at the 'complete' level, respectively. In order to ensure that each genome/sample belongs to the declared species, for each genome we estimated its Average Nucleotide Identity to the reference genome, samples that reached a value < 95% were discarded from the subsequent analysis. For each genome of a given species, we performed BLASTn against a randomly selected 16S rRNA sequence representing that species. Thus, we obtained the similarity of all copies within a given genome to the reference variant. We then focused on genomes in which we detected variability between individual copies of 16S rRNA. However, such genomes and variants then had to be thoroughly checked one by one, because the most common reason for excessively increased variability were deletion-affected sequences or usage of IUPAC degenerated symbols. We also partially used the clustering tool NanoCLUST to discover new variants**^17^**, however, mainly due to deletions, we used it on smaller preselected curated datasets instead of all 16S rRNA sequences. For these reasons, we also had to adopt very strict criteria for the classification of individual copies, whereby we considered a classification successful only if a given copy showed a 99.9% sequence identity with the reference variant.

In case we discovered true/legitimate alternative variants, we selected a set of representative genomes carrying only one of the detected variants and estimated a phylogenomic tree to determine if the variants correspond to certain phylogenetic sub-lineages, e.g. subspecies. Estimation of the phylogenomic tree was performed as described above, i.e. on the basis of protein sequences. The list of samples/genomes included in the phylogenomic analysis can be found in **Supplementary Table 3**. Additionally, we downloaded metadata such as submission date, source/host, location, and serotype for all genomes from NCBI using the Rentrez**^18^** library when these data were available. These data can be found in **Supplementary Table 4**.

**Supplementary References**

1. R Core Team. R: A language and environment for statistical computing. R Foundation for Statistical Computing, Vienna, Austria. [https://www.R-project.org/](https://www.r-project.org/) (2020).

2. Wickham, H. stringr:Simple, Consistent Wrappers for Common String Operations. R package version 1.4.0. [https://CRAN.R-project.org/package=stringr](https://cran.r-project.org/package=stringr) (2019).

3. Charif, D. & Lobry, J. R. SeqinR 1.0-2: A Contributed Package to the R Project for Statistical Computing Devoted to Biological Sequences Retrieval and Analysis. Structural Approaches to Sequence Evolution 207–232 (2007).

4. Altschul, S. F., Gish, W., Miller, W., Myers, E. W. & Lipman, D. J. Basic local alignment search tool. Journal of Molecular Biology vol. 215 403–410 (1990).

5. Lee, I., Ouk Kim, Y., Park, S.-C. & Chun, J. OrthoANI: An improved algorithm and software for calculating average nucleotide identity. International Journal of Systematic and Evolutionary Microbiology vol. 66 1100–1103 (2016).

6. Emms, D. M. & Kelly, S. OrthoFinder: phylogenetic orthology inference for comparative genomics. Genome Biology vol. 20 (2019).

7. Katoh, K. & Standley, D. M. MAFFT Multiple Sequence Alignment Software Version 7: Improvements in Performance and Usability. Molecular Biology and Evolution vol. 30 772–780 (2013).

8. Borowiec, M. L. AMAS: a fast tool for alignment manipulation and computing of summary statistics. PeerJ vol. 4 e1660 (2016).

9. Nguyen, L.-T., Schmidt, H. A., von Haeseler, A. & Minh, B. Q. IQ-TREE: A Fast and Effective Stochastic Algorithm for Estimating Maximum-Likelihood Phylogenies. Molecular Biology and Evolution vol. 32 268–274 (2014).

10. Hoang, D. T., Chernomor, O., von Haeseler, A., Minh, B. Q. & Vinh, L. S. UFBoot2: Improving the Ultrafast Bootstrap Approximation. Molecular Biology and Evolution vol. 35 518–522 (2017).

11. Kalyaanamoorthy, S., Minh, B. Q., Wong, T. K. F., von Haeseler, A. & Jermiin, L. S. ModelFinder: fast model selection for accurate phylogenetic estimates. Nature Methods vol. 14 587–589 (2017).

12. Mano, S. & Innan, H. The Evolutionary Rate of Duplicated Genes Under Concerted Evolution. Genetics vol. 180 493–505 (2008).

13. Mazerolle, M. J. _AICcmodavg: Model selection and multimodel inference based on (Q)AIC©_. R package version 2.3.3. https://CRAN.R-project.org/package=AICcmodavg (2023).

14. Chambers, J. M. *Linear models.* in *Statistical Models in S.* (eds. Hastie, T. J.), (Wadsworth & Brooks/Cole, 1992).

15. Best, D. J. & Roberts, D. E. Algorithm AS 89: The Upper Tail Probabilities of Spearman’s Rho. Applied Statistics vol. 24 377 (1975).

16. Liao, D. Concerted Evolution: Molecular Mechanism and Biological Implications. The American Journal of Human Genetics vol. 64 24–30 (1999).

17. Rodríguez-Pérez, H., Ciuffreda, L. & Flores, C. NanoCLUST: a species-level analysis of 16S rRNA nanopore sequencing data. Bioinformatics vol. 37 1600–1601 (2020).

18. Winter, D. J. (2017). rentrez: An R package for the NCBI eUtils API (No. e3179v2). PeerJ Preprints.
