# Supplementary material for: The overlooked evolutionary dynamics of 16S rRNA revises its role as the “gold standard” for bacterial species identification
Source: Sci Rep. 2024 Apr 20;14:9067. doi: 10.1038/s41598-024-59667-3 (PMC11032355; doi:10.1038/s41598-024-59667-3)
Supplement: Supplementary file 2 — Supplementary Figures. [file 41598_2024_59667_MOESM2_ESM.docx]

**Supplementary Figures**


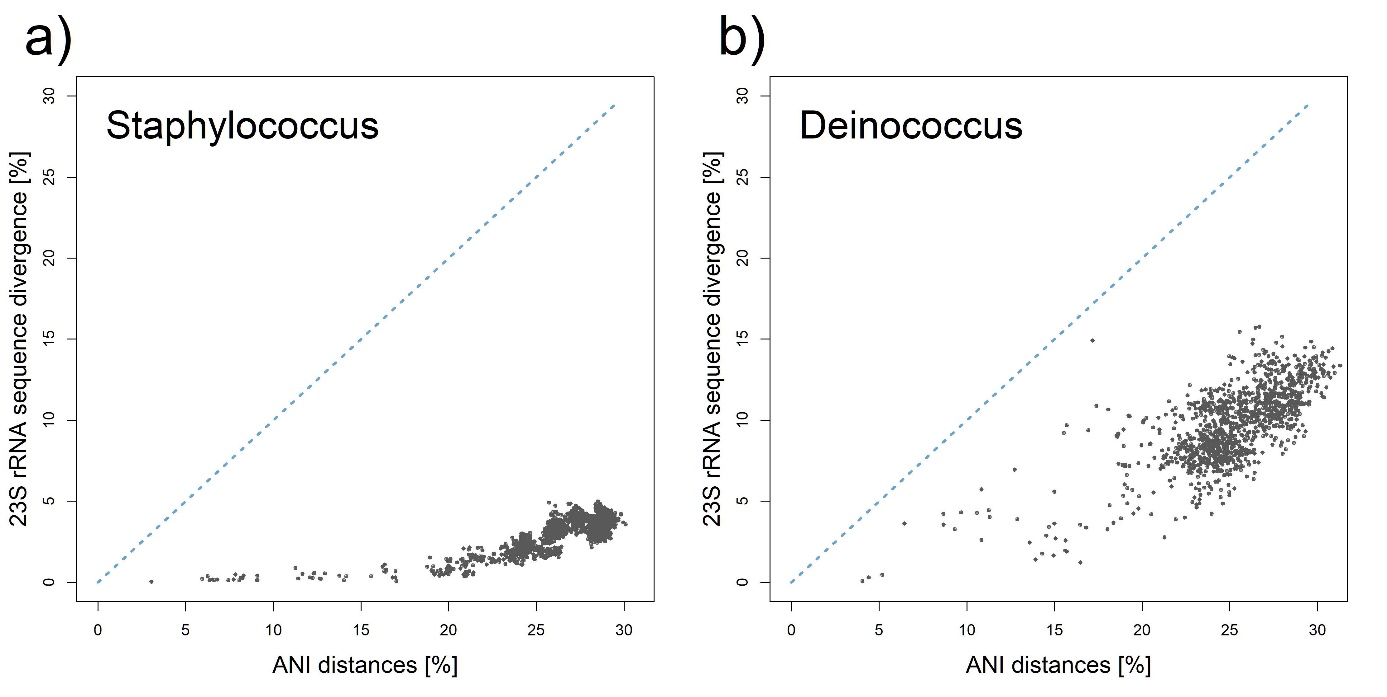


**Supplementary Figure 1**: Comparison of evolutionary (substitution) rates among studied bacterial genera at the level of 23S rRNA. The blue dashed line represents an idealized model of the sequence distance between the genomes of each species for a given value on the x-axis.


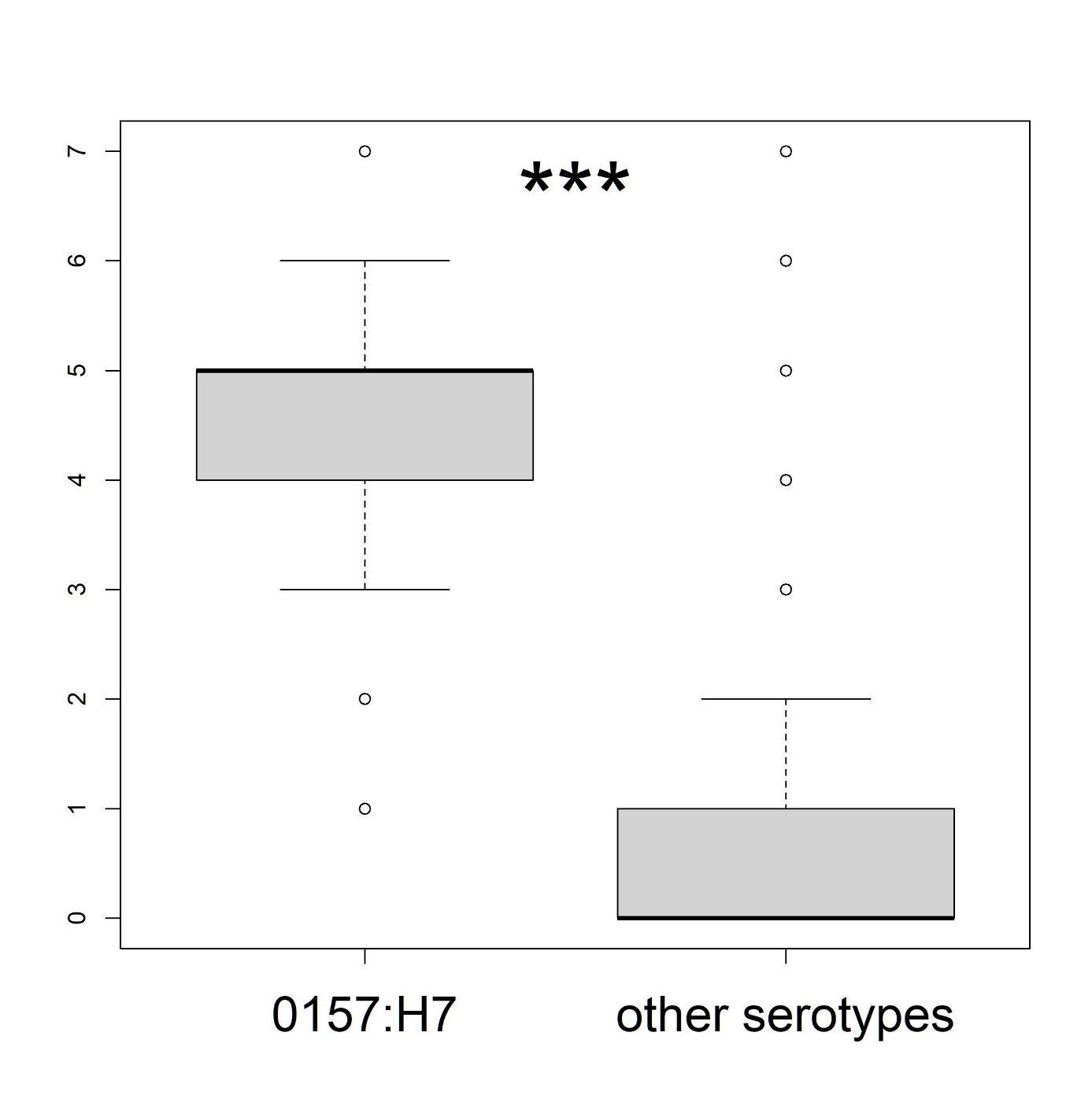


**Supplementary Figure 2**: Boxplot showing the copy number of a specific rssh 16S rRNA variant in *Escherichia coli* serotype 0157:H7 and in all other serotypes (i.e. excluding the properly labeled serotypes 0157:H7 and its predecessor 055:H7). Asterisks indicating the statistical significance of the difference between the two groups are based on the Wilcoxon Rank Sum test.

**
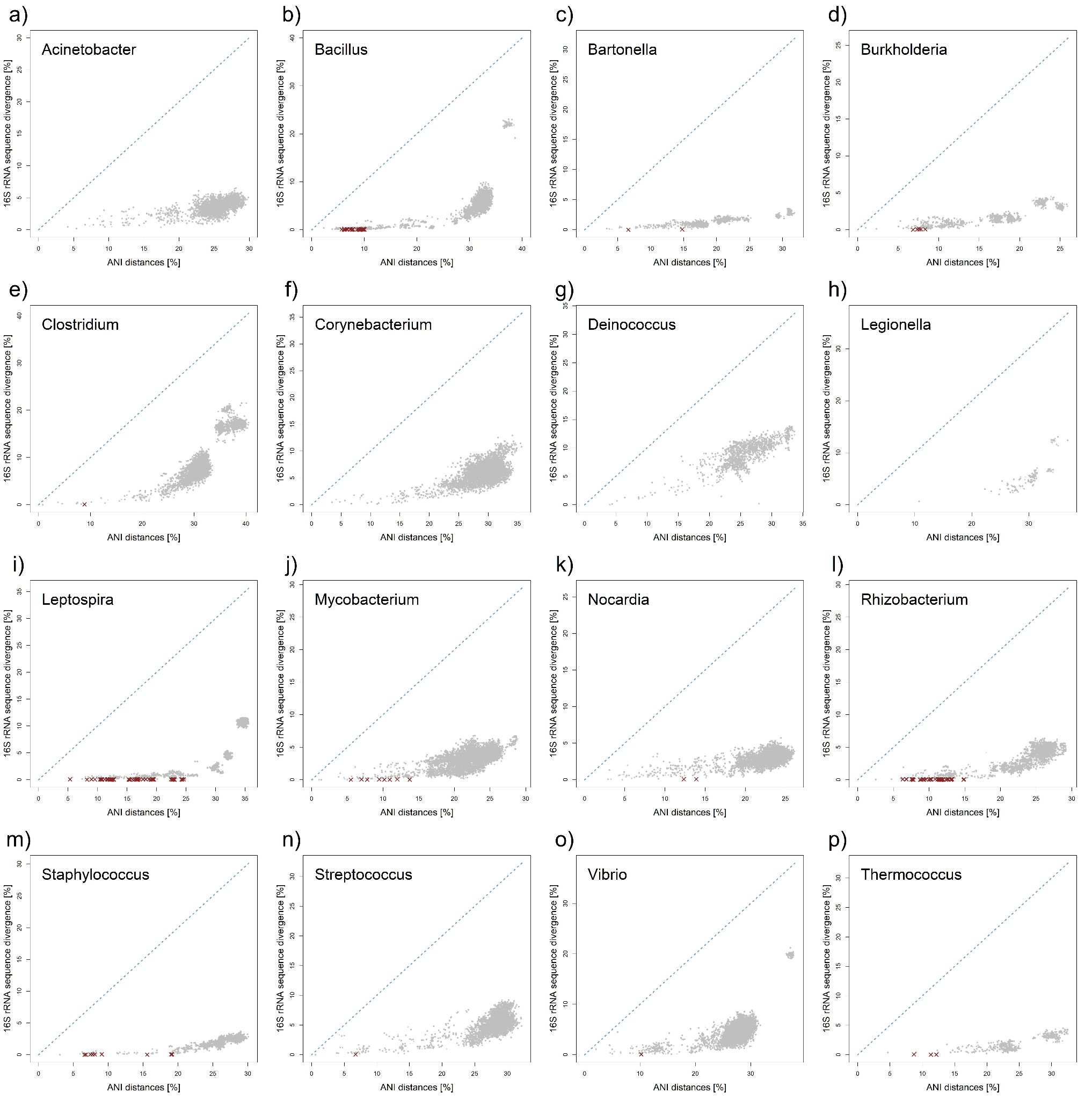
**

**Supplementary Figure 3**: Comparison of evolutionary (substitution) rates among studied bacterial genera. Individual comparisons are shown in grey, dark crosses indicate species pairs that share essentially the same copy of 16S rRNA (identity > 99.9%), despite being evolutionarily well-separated entities. The blue dashed line represents an idealized model of the sequence distance between the genomes of each species for a given value on the x-axis.

**
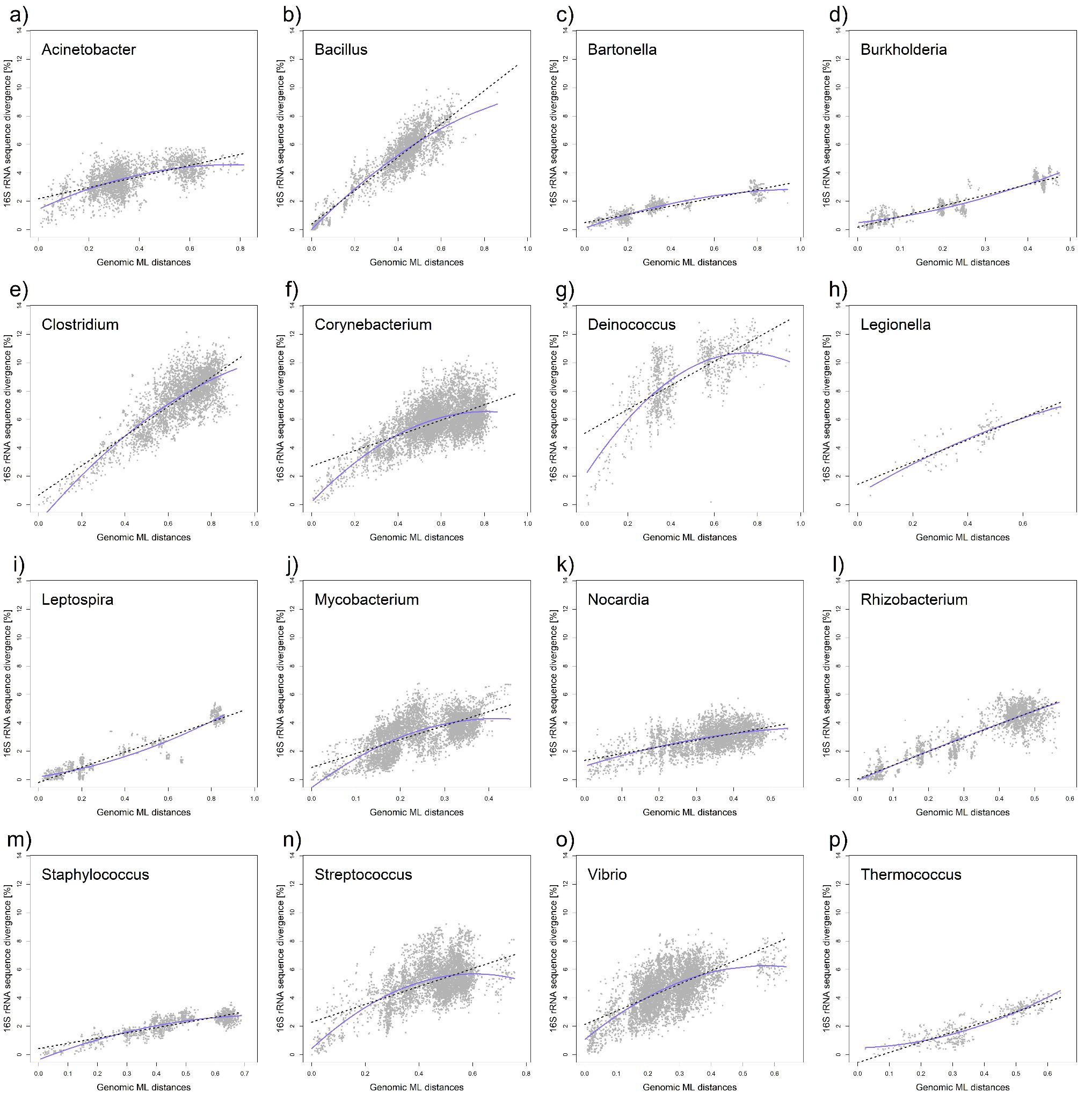
**

**Supplementary Figure 4**: Comparison of evolutionary (substitution) rates among studied bacterial genera. The black dashed line represents the fitted linear regression model, while the blue solid line represents the second-order polynomial function/model.


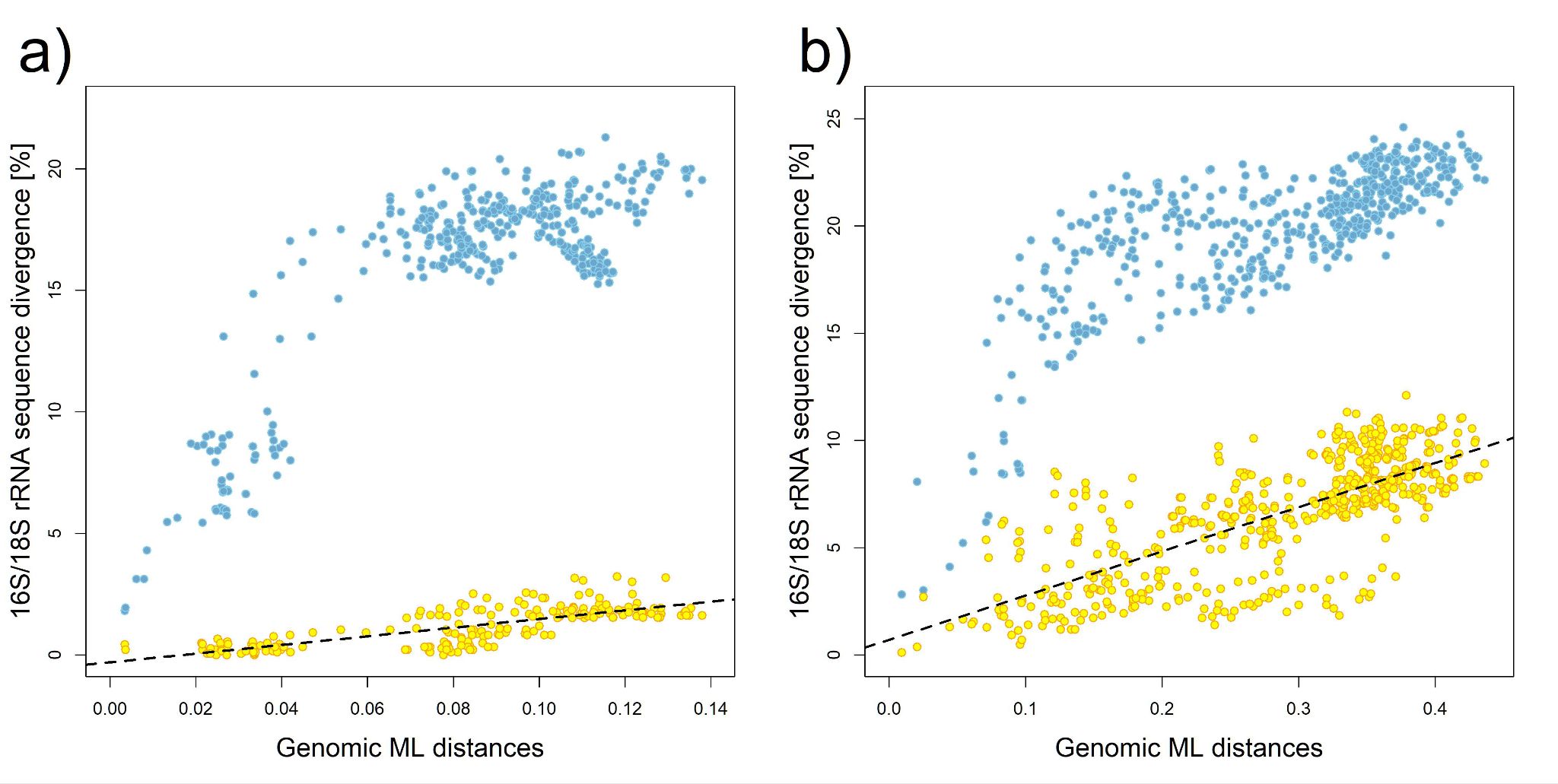


**Supplementary Figure 5**: Comparison of evolutionary (substituton) rates between 18S rRNA (yellow) and mito-ribosomal 16S rRNA (blue): **a)** Birds (Aves); **b)** Fish (Actinopterygii). The black dashed line represents the fitted linear regression model of 18S rRNA.
